# Supplementary material for: A fault-intrusion-tolerant system and deadline-aware algorithm for scheduling scientific workflow in the cloud
Source: PeerJ Comput Sci. 2021 Nov 2;7:e747. doi: 10.7717/peerj-cs.747 (PMC8576554; doi:10.7717/peerj-cs.747)
Supplement: Supplemental Information 6 [file peerj-cs-07-747-s006.zip › Myproject1.1/docs/index-files/index-1.html]

A-Index


JavaScript is disabled on your browser.


- Overview
- Package
- Class
- Use
- Tree
- Deprecated
- Index
- Help

- Prev Letter
- Next Letter

- Frames
- No Frames

- All Classes

A B C D E F G H I J L M N O P Q R S T U V W Z 


## A

ABRUPT\_END\_OF\_SIMULATION - Static variable in class org.cloudbus.cloudsim.core.CloudSimTags
:   Denotes an abrupt end of simulation.

abruptallyTerminate() - Static method in class org.cloudbus.cloudsim.core.CloudSim
:   Abruptally terminate.

abs(double[]) - Static method in class org.cloudbus.cloudsim.util.MathUtil
:   Abs.

AbstractArrayList - Class in org.workflowsim.clustering
:   This data structure AbstractArrayList is used in clustering alone It is
    better than ArrayList since it associates with depth information

AbstractArrayList(ArrayList, int) - Constructor for class org.workflowsim.clustering.AbstractArrayList
:   Initialize AbstractArrayList

accumulatedBwCost - Variable in class org.cloudbus.cloudsim.Cloudlet
:   The accumulated bw cost.

accuracy - Variable in class org.cloudbus.cloudsim.network.datacenter.AppCloudlet


addBaudRate(double) - Method in class org.cloudbus.cloudsim.InfoPacket
:   Every entity that the InfoPacket traverses should add the baud rate of the link on which this
    packet will be sent out next.

addChild(Task) - Method in class org.workflowsim.Task
:   Adds a task to existing child list

addChildList(List) - Method in class org.workflowsim.Task
:   Adds the list to existing child list

addClustDelay() - Method in class org.workflowsim.clustering.BasicClustering
:   For a clustered job, we should add clustering delay (by default it is
    zero)

addEntity(SimEntity) - Static method in class org.cloudbus.cloudsim.core.CloudSim
:   Add a new entity to the simulation.

addEntityDynamically(SimEntity) - Static method in class org.cloudbus.cloudsim.core.CloudSim
:   Internal method used to add a new entity to the simulation when the simulation is running.

addEntryTime(double) - Method in class org.cloudbus.cloudsim.InfoPacket
:   This method should be called by routers and other entities when this InfoPacket reaches them
    along with the current simulation time.

addEvent(SimEvent) - Method in class org.cloudbus.cloudsim.core.DeferredQueue
:   Adds a new event to the queue.

addEvent(SimEvent) - Method in class org.cloudbus.cloudsim.core.FutureQueue
:   Add a new event to the queue.

addEventFirst(SimEvent) - Method in class org.cloudbus.cloudsim.core.FutureQueue
:   Add a new event to the head of the queue.

addExitTime(double) - Method in class org.cloudbus.cloudsim.InfoPacket
:   This method should be called by routers and other entities when this InfoPacket is leaving
    them.

addFile(File) - Method in class org.cloudbus.cloudsim.Datacenter
:   Adds a file into the resource's storage before the experiment starts.

addFile(File) - Method in class org.cloudbus.cloudsim.HarddriveStorage
:   Adds a file to the storage.

addFile(List<File>) - Method in class org.cloudbus.cloudsim.HarddriveStorage
:   Adds a set of files to the storage.

addFile(File) - Method in class org.cloudbus.cloudsim.SanStorage
:   Adds a file to the storage.

addFile(List<File>) - Method in class org.cloudbus.cloudsim.SanStorage
:   Adds a set of files to the storage.

addFile(File) - Method in interface org.cloudbus.cloudsim.Storage
:   Adds a file to the storage.

addFile(List<File>) - Method in interface org.cloudbus.cloudsim.Storage
:   Adds a set of files to the storage.

addFile(File) - Method in class org.workflowsim.Task
:   Adds a file to existing file list

addHistoryEntry(HostDynamicWorkload, double) - Method in class org.cloudbus.cloudsim.power.PowerVmAllocationPolicyMigrationAbstract
:   Adds the history value.

addHop(int) - Method in class org.cloudbus.cloudsim.InfoPacket
:   This method should be called by network entities that count as hops, for e.g.

addLink(TopologicalLink) - Method in class org.cloudbus.cloudsim.network.TopologicalGraph
:   adds an link between two topological nodes

addLink(int, int, double, double) - Static method in class org.cloudbus.cloudsim.NetworkTopology
:   Adds a new link in the network topology

addLocalFile(File) - Method in class org.workflowsim.CondorVM
:   Adds a file to the local file system

addMigratingInVm(Vm) - Method in class org.cloudbus.cloudsim.Host
:   Adds the migrating in vm.

addNode(TopologicalNode) - Method in class org.cloudbus.cloudsim.network.TopologicalGraph
:   adds an Topological Node to this graph

addParent(Task) - Method in class org.workflowsim.Task
:   Adds a task to existing parent list

addParentList(List) - Method in class org.workflowsim.Task
:   Adds the list to existing parent list

addRequiredFile(String) - Method in class org.cloudbus.cloudsim.Cloudlet
:   Adds the required filename to the list.

addReservedFile(File) - Method in class org.cloudbus.cloudsim.HarddriveStorage
:   Adds a file for which the space has already been reserved.

addReservedFile(File) - Method in class org.cloudbus.cloudsim.SanStorage
:   Adds a file for which the space has already been reserved.

addReservedFile(File) - Method in interface org.cloudbus.cloudsim.Storage
:   Adds a file for which the space has already been reserved.

addRoot() - Method in class org.workflowsim.clustering.BasicClustering


addStateHistoryEntry(double, double, double, boolean) - Method in class org.cloudbus.cloudsim.HostDynamicWorkload
:   Adds the state history entry.

addStateHistoryEntry(double, double, double, boolean) - Method in class org.cloudbus.cloudsim.Vm
:   Adds the state history entry.

addStorageList(String, String) - Static method in class org.workflowsim.utils.ReplicaCatalog
:   Adds a file to a storage

addTask(Task) - Method in class org.workflowsim.clustering.TaskSet
:   Adds a task to this taskSet

addTask(ArrayList<Task>) - Method in class org.workflowsim.clustering.TaskSet
:   Adds a list of task to this taskSet

addTaskList(List) - Method in class org.workflowsim.Job
:   Adds a task list to the existing task list

addTasks2Job(Task) - Method in class org.workflowsim.clustering.BasicClustering
:   Add a task to a new job

addTasks2Job(List) - Method in class org.workflowsim.clustering.BasicClustering
:   Add a list of task to a new job

addTaskSet2TaskSet(TaskSet, TaskSet) - Method in class org.workflowsim.clustering.balancing.methods.BalancingMethod
:   Add all the tasks in tail to head and then clean tail.

addUtilizationHistoryValue(double) - Method in class org.cloudbus.cloudsim.power.PowerVm
:   Adds the utilization history value.

ADVANCE\_RESERVATION - Static variable in class org.cloudbus.cloudsim.DatacenterCharacteristics
:   A resource that supports Advanced Reservation mechanisms.

Agg\_LEVEL - Static variable in class org.cloudbus.cloudsim.network.datacenter.NetworkConstants


AggregateSwitch - Class in org.cloudbus.cloudsim.network.datacenter
:   This class allows to simulate aggregate switch for Datacenter network.

AggregateSwitch(String, int, NetworkDatacenter) - Constructor for class org.cloudbus.cloudsim.network.datacenter.AggregateSwitch
:   Constructor for Aggregate Switch We have to specify switches that are connected to its
    downlink and uplink ports, and corresponding bandwidths

AggSwitchPort - Static variable in class org.cloudbus.cloudsim.network.datacenter.NetworkConstants


allocateBwForVm(Vm, long) - Method in class org.cloudbus.cloudsim.provisioners.BwProvisioner
:   Allocates BW for a given VM.

allocateBwForVm(Vm, long) - Method in class org.cloudbus.cloudsim.provisioners.BwProvisionerSimple


allocateHostForVm(Vm) - Method in class org.cloudbus.cloudsim.network.datacenter.NetworkVmAllocationPolicy
:   Allocates a host for a given VM.

allocateHostForVm(Vm, Host) - Method in class org.cloudbus.cloudsim.network.datacenter.NetworkVmAllocationPolicy


allocateHostForVm(Vm) - Method in class org.cloudbus.cloudsim.power.PowerVmAllocationPolicyAbstract


allocateHostForVm(Vm, Host) - Method in class org.cloudbus.cloudsim.power.PowerVmAllocationPolicyAbstract


allocateHostForVm(Vm) - Method in class org.cloudbus.cloudsim.VmAllocationPolicy
:   Allocates a host for a given VM.

allocateHostForVm(Vm, Host) - Method in class org.cloudbus.cloudsim.VmAllocationPolicy
:   Allocates a specified host for a given VM.

allocateHostForVm(Vm) - Method in class org.cloudbus.cloudsim.VmAllocationPolicySimple
:   Allocates a host for a given VM.

allocateHostForVm(Vm, Host) - Method in class org.cloudbus.cloudsim.VmAllocationPolicySimple


allocateMipsForVm(Vm, double) - Method in class org.cloudbus.cloudsim.provisioners.PeProvisioner
:   Allocates MIPS for a given VM.

allocateMipsForVm(String, double) - Method in class org.cloudbus.cloudsim.provisioners.PeProvisioner
:   Allocates MIPS for a given VM.

allocateMipsForVm(Vm, List<Double>) - Method in class org.cloudbus.cloudsim.provisioners.PeProvisioner
:   Allocates MIPS for a given VM.

allocateMipsForVm(Vm, double) - Method in class org.cloudbus.cloudsim.provisioners.PeProvisionerSimple


allocateMipsForVm(String, double) - Method in class org.cloudbus.cloudsim.provisioners.PeProvisionerSimple


allocateMipsForVm(Vm, List<Double>) - Method in class org.cloudbus.cloudsim.provisioners.PeProvisionerSimple


allocatePesForVm(Vm, List<Double>) - Method in class org.cloudbus.cloudsim.Host
:   Allocates PEs for a VM.

allocatePesForVm(Vm, List<Double>) - Method in class org.cloudbus.cloudsim.VmScheduler
:   Allocates PEs for a VM.

allocatePesForVm(Vm, List<Double>) - Method in class org.cloudbus.cloudsim.VmSchedulerSpaceShared


allocatePesForVm(Vm, List<Double>) - Method in class org.cloudbus.cloudsim.VmSchedulerTimeShared


allocatePesForVm(String, List<Double>) - Method in class org.cloudbus.cloudsim.VmSchedulerTimeShared
:   Allocate pes for vm.

allocatePesForVm(String, List<Double>) - Method in class org.cloudbus.cloudsim.VmSchedulerTimeSharedOverSubscription
:   Allocate pes for vm.

allocateRamForVm(Vm, int) - Method in class org.cloudbus.cloudsim.provisioners.RamProvisioner
:   Allocates RAM for a given VM.

allocateRamForVm(Vm, int) - Method in class org.cloudbus.cloudsim.provisioners.RamProvisionerSimple


allPairsShortestPaths(float[][]) - Method in class org.cloudbus.cloudsim.network.FloydWarshall\_Float
:   calculates all pairs delay

allTaskNum - Variable in class org.workflowsim.failure.FailureRecord
:   all the tasks (failed or not).

analyze(int, int) - Static method in class org.workflowsim.failure.FailureMonitor
:   Update the detected task failure rate based on record lists

APP\_MC - Static variable in class org.cloudbus.cloudsim.network.datacenter.AppCloudlet


APP\_Workflow - Static variable in class org.cloudbus.cloudsim.network.datacenter.AppCloudlet


AppCloudlet - Class in org.cloudbus.cloudsim.network.datacenter
:   AppCloudlet class represents an application which user submit for execution within datacenter.

AppCloudlet(int, int, double, int, int) - Constructor for class org.cloudbus.cloudsim.network.datacenter.AppCloudlet


appID - Variable in class org.cloudbus.cloudsim.network.datacenter.AppCloudlet


areAllVmsMigratingOutOrAnyVmMigratingIn(PowerHost) - Method in class org.cloudbus.cloudsim.power.PowerVmAllocationPolicyMigrationAbstract
:   Checks whether all vms are in migration.

ArgumentParser - Class in org.workflowsim.utils
:   This class implements ArgumentParser that parse arguments and set the
    parameters

ArgumentParser(String[]) - Constructor for class org.workflowsim.utils.ArgumentParser
:   This method initialize a ArgumentParser object

A B C D E F G H I J L M N O P Q R S T U V W Z

- Overview
- Package
- Class
- Use
- Tree
- Deprecated
- Index
- Help

- Prev Letter
- Next Letter

- Frames
- No Frames

- All Classes
